# Harnessing Decellularized Extracellular Matrix for Enhanced Fidelity in Colorectal Cancer Organoid and Cell-Derived Xenograft Models

**DOI:** 10.4014/jmb.2405.05036

**Published:** 2024-06-27

**Authors:** Yena Nam, Eunju Cha, Su Min Kwak, Seung Ju Seo, John Hoon Rim, Yoonhee Jin

**Affiliations:** 1 Department of Physiology, Graduate School of Medical Science, Brain Korea 21 Project, Yonsei University College of Medicine, Seoul 03722, Republic of Korea; 2 Department of Medicine, College of Medicine, Yonsei University Graduate School, Seoul 03722, Republic of Korea; 3 Department of Laboratory Medicine, Yonsei University College of Medicine, Seoul, Republic of Korea

**Keywords:** Patient-derived tumor organoid, colorectal cancer, extracellular matrix, xenograft models

## Abstract

This study evaluates the efficacy of a decellularized intestine tissue-derived extracellular matrix (Intestine ECM) as a scaffold for culturing colorectal cancer (CRC) organoids and establishing cell-derived xenograft (CDX) models, comparing its performance to traditional Matrigel. Intestine ECM demonstrates comparable support for organoid formation and cellular function, highlighting its potential as a more physiologically relevant and reproducible platform. Our findings suggest that Intestine ECM enhances the mimetic environment for colon epithelium, supporting comparable growth and improved differentiation compared to Matrigel. Moreover, when used as a delivery carrier, Intestine ECM significantly increases the growth rate of CDX models using patient-derived primary colorectal cancer cells. This enhancement demonstrates Intestine ECM’s role not only as a scaffold but also as a vital component of the tumor microenvironment, facilitating more robust tumorigenesis. These findings advocate for the broader application of Intestine ECM in cancer model systems, potentially leading to more accurate preclinical evaluations and the development of targeted cancer therapies.

## Introduction

The advent of organoid technology has marked a significant shift in biological and biomedical research, enabling the in vitro recapitulation of complex, organ-specific architectures, and functions from simple two-dimensional (2D) cell cultures [1, 2]. Organoids, three-dimensional (3D) constructs that mimic miniature organs, have become pivotal in studying organ development, disease mechanisms, and applications in drug discovery and regenerative medicine. Among these, patient-derived tumor organoids (PDOs) represent a substantial advancement, bridging the gap between traditional immortalized cell lines and patient-derived xenograft (PDX) models [3-5]. Notably, PDOs preserve the histological features, gene expression profiles, and drug responses of the donor tumors, establishing them as reliable models for preclinical evaluations of anticancer agents and personalized cancer therapies [6-8].

Organoids require precise cultivation under defined conditions, including specific growth factors and a supportive scaffold, to self-organize into structures that closely mimic their in vivo counterparts [9-11]. This fidelity is vital for cancer research, where replicating patient-specific tumor characteristics and heterogeneity is crucial [12-14]. Traditionally, Matrigel, a hydrogel derived from the Engelbreth-Holm-Swarm mouse sarcoma, has been the standard scaffold for organoid culture. Although its composition mirrors that of the natural extracellular matrix (ECM), containing essential proteins like laminin, collagen IV, entactin, and heparan sulfate proteoglycans necessary for 3D growth, its non-tissue-specific origin presents significant limitations [15,16]. This is particularly critical in colorectal cancer, where the ECM is extensively remodeled and disorganized, playing a pivotal role in influencing key cancer hallmarks such as differentiation, proliferation, angiogenesis, invasion, and metastasis [17-19].

In response to these challenges, tissue decellularization processes have given rise to promising alternatives [20, 21]. These decellularized tissue-derived ECMs (dECMs) preserve a complex array of ECM proteins, growth factors, and other bioactive molecules inherent to the source organ [22, 23]. Commercially available as Regenix® Intestine, derived from porcine intestine, is one such dECM, facilitating an organ-specific microenvironment that supports more effective organoid growth and differentiation than synthetic alternatives [25]. This study utilizes decellularized intestine tissue-derived extracellular matrix (Intestine ECM), to explore its potential as a scaffold for cultivating human colorectal cancer (CRC) organoids and establishing patient cell-derived xenograft (CDX) mouse models. By leveraging this specific ECM, the research aims to enhance the physiological relevance and reproducibility of our models, potentially improving our understanding of disease mechanisms and aiding in developing more effective therapeutic interventions.

## Materials and Methods

### Cell Culture

This study, involving human colon cancer cells and CRC organoids isolated from patients, was approved by the Institutional Review Board (IRB) of Yonsei University Health System (Permit Number: 4-2023-1545). The CRC organoids (#SNU4351S4-4-TO, #SNU4646S1-TO) were sourced from the Korean Cell Line Bank (Republic of Korea). For CRC organoid culture, the medium consisted of Advanced DMEM/F12 (Dulbecco’s Modified Eagle Medium/HAM’s F-12; #12634-010, Thermo Fisher Scientific, USA) supplemented with 10 mM HEPES (#15630-080, Thermo Fisher Scientific), 2 mM Glutamax (#35050061, Thermo Fisher Scientific), 1% (v/v) penicillin-streptomycin (P/S; #15140122, Thermo Fisher Scientific), 1 mM N-acetyl cysteine (#A9165, Sigma-Aldrich, USA), 0.5 μM A83-01 (#2393, Tocris Bioscience, UK), 1 × B27 (#17504044, Thermo Fisher Scientific), 10 μM SB202190 (#S7067, Sigma-Aldrich), 10 nM gastrin I (#G9145, Sigma- Aldrich), and 50 ng/ml *Epidermal Growth Factor* (*EGF*) (#AF-100-15, Peprotech, USA). 10 μM Y-27632 (#1293823, Biogems International, Inc., USA) was added to the medium during the first 1-2 days following passaging. For passaging CRC organoids, Matrigel (#354230, Corning, USA) was degraded using Cell Recovery Solution (#354253, Corning) at 4°C for one hour. For passaging organoids cultured in Intestine ECM hydrogel (#Regenix Intestine, Cellartgen, Korea)—a commercially available decellularized extracellular matrix derived from porcine intestine—were degraded with 2 mg/ml collagenase IV (#17104019, Thermo Fisher) at 37°C for one hour. The colon cancer cell line, SNU-C2B (Korean Cell Line Bank), was cultured in Roswell Park Memorial Institute (RPMI)-1640 medium (#11875093, Thermo Fisher Scientific), supplemented with 10% heat-inactivated fetal bovine serum (#26140079, Thermo Fisher Scientific), 1% (v/v) P/S, and 25 mM HEPES.

### Immunofluorescence Staining

Immunofluorescence staining was performed on CRC organoids cultured for 7 days. Initially, Matrigel and Intestine ECM hydrogels were degraded using either Cell Recovery Solution or 2 mg/ml collagenase IV, respectively. The organoids were then fixed in 4% paraformaldehyde (Sigma-Aldrich) for one hour, permeabilized with 0.2% Triton X-100 (Sigma-Aldrich) for 30 min and blocked in 5% bovine serum albumin for 2 h at room temperature (RT). Subsequently, organoids were incubated with primary antibodies, including rabbit anti-Ki67 (#ab15580, 1:1000, Abcam, UK), rabbit anti-mucin 2 (MUC2; #sc-15334, 1:200, Santa Cruz Biotechnology, USA), mouse-anti-E-cadherin (#610181, BD Bioscience, USA) for 24 h at 4°C. After washing three times with phosphate-buffered saline (PBS), the organoids were exposed to secondary antibodies conjugated with goat anti-rabbit Alexa-Fluor 594 (#A-11012, 1:200, Thermo Fischer Scientific), or goat anti-mouse IgG Alexa-Fluor 488 (#A-11008, 1:200, Thermo Fischer Scientific) for another 24 h at 4°C. Filamentous-actin (F-actin) staining was performed with Phalloidin-iFluor 488 Reagent (#ab176753, 1:1000, Abcam) for 24 h at 4°C. After multiple washes with PBS, nuclei were stained with 2-(4-amidinophenyl)-6-indolecarboxamidine dihydrochloride (DAPI, TCI, Japan) for 30 min at RT. The immunostained organoids were visualized using a LSM 980 confocal microscope (Carl Zeiss, Germany).

### Quantitative Reverse Transcription Polymerase Chain Reaction (qRT-PCR)

The mRNA expression profiles of CRC organoids were assessed using qPCR. mRNA samples were extracted from organoids cultured for 9 days using the TaKaRa miniBEST Universal RNA Extraction Kit (TaKaRa, Japan). cDNA was then synthesized from the isolated mRNA using a cDNA synthesis kit (TaKaRa). qPCR was performed using the TaqMan Fast Universal Master Mix system (Applied Biosystems, USA) on a QuantStudio 3 Real-Time PCR system (Applied Biosystems). Primers targeting leucine-rich repeat-containing G-protein coupled receptor 5 (*LGR5*; Hs00969422_m1), Ki67 (*MKI67*; Hs04260396_g1), mucin 2 (*MUC2*; Hs03005103_g1), and villin (*VILL*; Hs00210626_m1) were used. Relative gene expression levels were quantified using the cycle threshold (Ct) method, with glyceraldehyde 3-phosphate dehydrogenase (*GAPDH*; Hs02786624_g1) serving as the reference gene.

### Forskolin-Induced Swelling Assay

Forskolin-induced swelling assays were conducted on CRC organoids to assess the regulation of luminal fluid secretion [24, 25]. Organoids cultured in either Intestine ECM or Matrigel were incubated in culture medium supplemented with 10 μM forskolin. The organoid size was measured every 20 min using an EVOS M5000 microscope (Thermo Fisher Scientific). Area analysis of the organoids was conducted using ImageJ software (National Institutes of Health, USA).

### Animals and Ethics Statements

For in vivo studies, the procedures were approved by the Institutional Animal Care and Use Committee of Yonsei University College of Medicine under permit number 2024-0106. All experiments involving animals were conducted in facilities accredited by the Association for Assessment and Accreditation of Laboratory Animal Care International (AAALAC International). The animals were maintained under controlled environmental conditions with a temperature of 21 ± 2°C, humidity of 50 ± 10%, ventilation of 10-15 air changes per hour, and noise levels maintained below 60 dB.

### Generation of CDX Models

Colon cancer cells (#SNU-C2B) were used for engraftment into 6-week-old BALB/c nude male mice (Orientbio, Republic of Korea). The nude mouse was anesthetized with 2% inhaled isoflurane. Each mouse received a subcutaneous injection of 1 × 10^6^ cells in a 100 μl Intestine ECM (#Regenix Intestine) into the right flank. For the control group, cells resuspended in 100 μl PBS was injected.

## Results

### Characterization of CRC Organoids Cultured in Intestine ECM

**Fig. 1:**
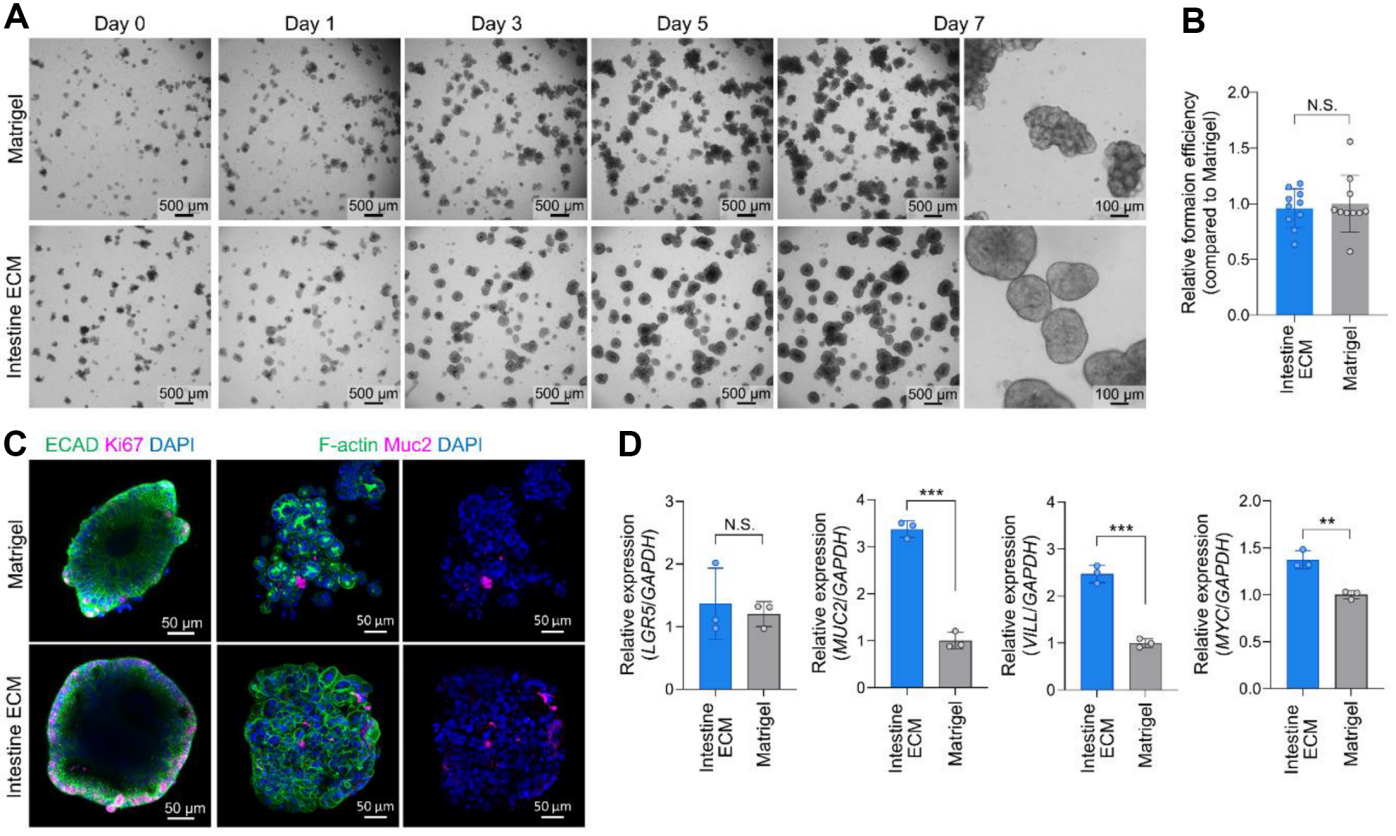
CRC organoid cultivation in Intestine ECM demonstrates enhanced differentiation. (**A**) Bright-field images showing the progression of CRC organoids from day 0 to day 7 cultured in Intestine ECM and Matrigel (Scale bars = 100 μm and 500 μm), demonstrating organoid growth over time. (**B**) Quantification of organoid formation efficiency in Intestine ECM compared to Matrigel (*n* = 10, statistical analysis performed using a two-tailed unpaired *t*-test). (**C**) Immunofluorescence staining images for ECAD (intestinal epithelial marker), Ki67 (proliferation marker), Muc2 (goblet cell differentiation marker), and F-actin (cytoskeleton staining) of CRC organoids cultured in Intestine ECM and Matrigel (scale bars = 50 μm), illustrating cellular composition and architecture. (**D**) Gene expression analysis of key markers between organoids cultured in Intestine ECM and Matrigel (*n* = 3). Data analyzed using a two-tailed unpaired *t*-test; significance indicated by *p < 0.05, **p < 0.01, ***p < 0.001 versus Matrigel group, highlighting differences in gene expression levels.

In assessing the effectiveness of the Intestine ECM for CRC organoid cultures, we compared it directly to the traditionally used Matrigel. Our findings revealed that organoid formation efficiency was comparably high with both Intestine ECM and Matrigel (Fig. 1A and 1B), demonstrating that the biophysical and biochemical properties of Intestine ECM are adequate for supporting the growth and maintenance of CRC organoids.

Immunostaining revealed that the intestinal epithelial marker (ECAD), the proliferation marker (Ki67), and the goblet cell marker (MUC2) crucial for the colonic mucus barrier [26, 27], exhibited similar expression levels in organoids cultured in both Intestine ECM and Matrigel (Fig. 1C). This similarity indicates the presence of actively dividing cells and suggests that Intestine ECM supports cellular proliferation effectively. Furthermore, no significant differences were observed in the number of focal adhesions or the organization of the cytoskeleton between the two scaffolds, indicating that Intestine ECM maintains cell-matrix interactions comparable to those of Matrigel (Fig. 1C).

Further investigation was carried out through qRT-PCR analysis (Fig. 1D). The analysis revealed that the stemness marker *LGR5* in CRC organoids expressed at comparable levels in both scaffolds, confirming that Intestine ECM supports stem cell populations effectively. Importantly, differentiation markers such as *MUC2* and *VILL*, an enterocyte marker gene [28, 29], showed significantly higher expression in organoids cultured in Intestine ECM. This increase in differentiation marker expression suggests that Intestine ECM provides an enhanced mimetic environment for colon epithelial biology compared to Matrigel. Additionally, the proto-oncogene *MYC*, known to be associated with cancer progression [30-32], was also elevated in the Intestine ECM scaffold, further indicating the scaffold's potential to support enhanced intestinal gene expression.

### Consistency across Different Batches of Intestine ECM

**Fig. 2:**
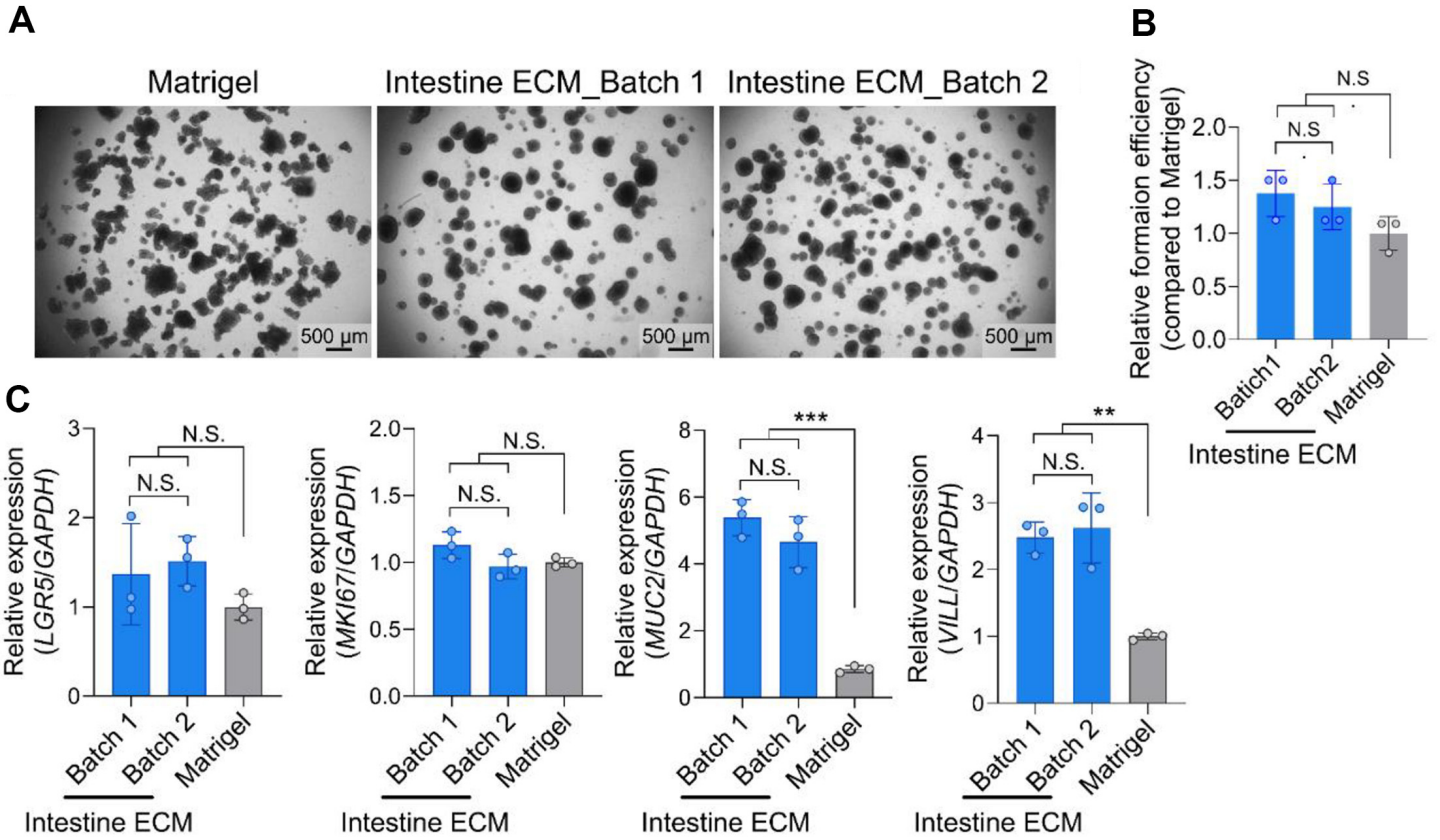
Consistent performance of Intestine ECM across different batches for culturing CRC organoids. (**A**) Bright-field images of CRC organoids cultured in Intestine ECM from different production lots of Intestine ECM after 7 days (scale bars = 500 μm), illustrating the uniformity in organoid growth and morphology. (**B**) Quantification of organoid formation efficiency in two different batches of Intestine ECM compared to Matrigel (*n* = 3), illustrating consistent performance across batches. (*n* = 3). (**C**) Gene expression analysis comparing two different batches of Intestine ECM (*n* = 3). Data were analyzed using one-way ANOVA with a Tukey multiple comparison test. No significant differences were found, indicated by N.S. (not significant); **p < 0.01, ***p < 0.001 versus Matrigel group.

Given the natural origin of dECM products, concerns about batch-to-batch variability are significant. Addressing this, CRC organoids were cultured using Intestine ECM sourced from different production lots (Fig. 2). The findings revealed consistent organoid formation efficiencies across two distinct batches of Intestine ECM (Fig. 2A and 2B). Additionally, similar expression levels of proliferation and intestinal markers, including *LGR5*, *MKI67*, *MUC2*, and *VILL* were observed (Fig. 2C). This consistency in performance indicates the reliability of Intestine ECM as a scaffold and suggests that, despite its derivation from natural sources, Intestine ECM maintains stable quality across different production lots.

### Functional Assessment of CFTR in CRC Organoids Cultured in Intestine ECM

**Fig. 3:**
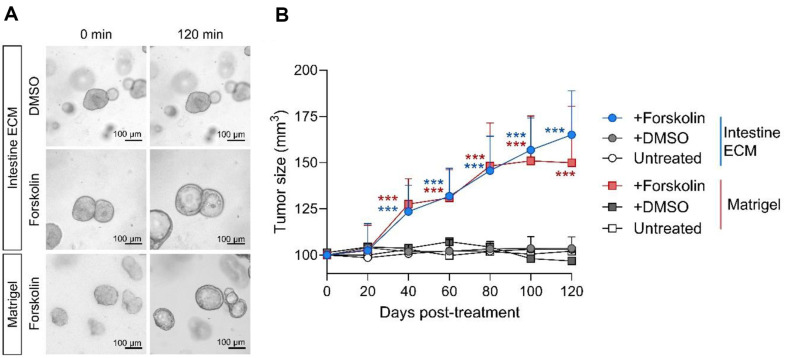
Comparable forskolin-induced swelling in CRC organoids cultured in Intestine ECM. (**A**) Bright-field images showing CRC organoids before and 120 min after treatment with 10 μM forskolin, demonstrating the organoids' enlargement (scale bars = 100 μm). (**B**) Quantification of the rate of enlargement measured at 20-min intervals up to 120 min. The increase in size is significant compared to organoids with no treatment and those treated with a DMSO control (*n* = 11). Data analyzed using two-way ANOVA with Tukey's multiple comparison test. ***P < 0.001 versus respective DMSO control groups (asterisks in blue for Intestine ECM, in red for Matrigel).

Next, the function of the cystic fibrosis transmembrane conductance regulator (CFTR) in CRC organoids cultured in Intestine ECM hydrogel was compared with those cultured in Matrigel, utilizing a forskolin-induced swelling assay (Fig. 3). Both groups of CRC organoids exhibited rapid growth at similar rates following forskolin treatment, indicating that organoids cultivated in Intestine ECM hydrogel maintain their ability to regulate luminal fluid secretion (Fig. 3A and 3B). These results validate the effectiveness of Intestine ECM hydrogels as platforms for PDO culture, supporting the generation and differentiation of CRC organoids with structural and functional characteristics comparable to those grown in Matrigel.

### Generation of Xenograft Models Using Intestine ECM

**Fig. 4:**
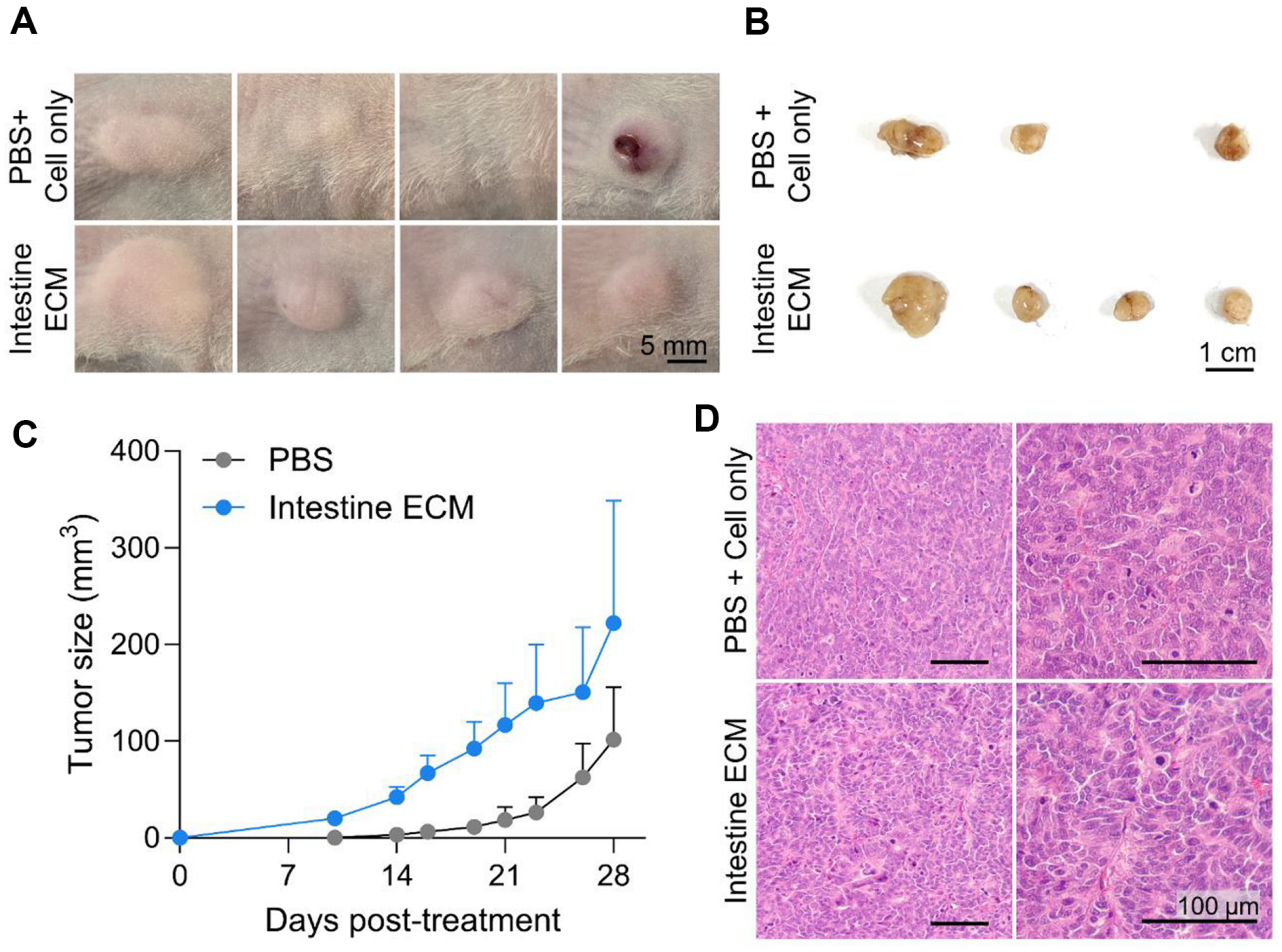
Efficacy of Intestine ECM in supporting colon cancer CDX model development. (**A**) Photographs of the tumor on mice at 28 days post-injection of colon cancer cells with Intestine ECM (scale bar = 5 mm). (**B**) Images of the tumor after extraction, demonstrating size and morphology (scale bar = 1 cm). (**C**) Graph showing the growth trajectory of tumors over time, with measurements taken at regular intervals after injection of CRCs mixed with Intestine ECM. This illustrates the progressive increase in tumor size, indicating effective tumor development. (**D**) Hematoxylin and eosin (H&E) stained images of sectioned tumors (scale bars = 100 μm).

To assess the efficacy of Intestine ECM in supporting tumor formation, we utilized primary patient-derived colorectal cancer cells, which were injected into immunodeficient mice to form xenograft models (Fig. 4). Over a period of four weeks, cells injected with Intestine ECM demonstrated a significantly faster rate of tumor formation compared to the control where only cells were injected (Fig. 4A). Tumors formed with Intestine ECM were significantly larger in volume, indicating enhanced tumorigenic potential (Fig. 4A-4C). Histological examination using hematoxylin and eosin (H&E) staining revealed similar morphological characteristics between the two groups (Fig. 4D).

## Discussion

This study has successfully demonstrated the effectiveness of Intestine ECM as a scaffold for CRC organoids and establishing CDX models. By showing that Intestine ECM supports organoid formation and cellular functions as competently as Matrigel, this study makes a significant contribution to the field of oncology research. Intestine ECM provides a more physiologically relevant and reproducible platform, which is crucial for improving the accuracy of preclinical models in predicting clinical outcomes and facilitating the development of personalized medicine approaches.

One of the core advantages of using patient-derived cancer organoids is their ability to maintain the genetic and phenotypic characteristics of the donor's tumor, providing a unique opportunity to tailor chemotherapy options to individual patients. These organoids serve as a vital tool for oncologists, enabling the customization of treatment plans based on the specific cellular behavior exhibited by the organoid model. By integrating Intestine ECM in the cultivation of these organoids, our study leverages this capability to simulate a more accurate tumor microenvironment, enhancing the relevance of our findings to actual clinical scenarios.

The application of PDX models, crucial for preclinical evaluations of anticancer drugs, benefits significantly from advancements in scaffold technologies like Intestine ECM. PDX models, which maintain the histopathological and genetic characteristics of human tumors, serve as essential tools for screening molecular markers and testing drug responses. However, the variability in tumor engraftment efficacy among different cell lines underscores the need for improved methodologies like those provided by Intestine ECM, which has shown potential to accelerate tumor growth and increase tumor volume. By enhancing the engraftment success rate and tumor dynamics, Intestine ECM could substantially improve the utility of PDX models in drug testing and development. Our use of comprehensive and robust methodologies in this study establishes the effectiveness of Intestine ECM in creating accurate and clinically relevant models of colorectal cancer. These models reflect the complex interactions within the tumor microenvironment and offer substantial promise for advancing personalized cancer therapy.
